# Decomposition of Calcium Oxalate Crystals in *Colobanthus quitensis* under CO_2_ Limiting Conditions

**DOI:** 10.3390/plants9101307

**Published:** 2020-10-02

**Authors:** Olman Gómez-Espinoza, Daniel González-Ramírez, Panagiota Bresta, George Karabourniotis, León A. Bravo

**Affiliations:** 1 Laboratorio de Fisiología y Biología Molecular Vegetal, Instituto de Agroindustria, Departamento de Ciencias Agronómicas y Recursos Naturales, Facultad de Ciencias Agropecuarias y Forestales & Center of Plant, Soil Interaction and Natural Resources Biotechnology, Scientific and Technological Bioresource Nucleus, Universidad de La Frontera, 1145 Temuco, Chile; o.gomez01@ufromail.cl or; 2 Centro de Investigación en Biotecnología, Escuela de Biología, Instituto Tecnológico de Costa Rica, Cartago 30101, Costa Rica; daniel.agr13@estudiantec.cr; 3 Laboratory of Plant Physiology and Morphology, Faculty of Crop Science, Agricultural University of Athens, 118 55 Athens, Greece; brestapan@aua.gr (P.B.); karab@aua.gr (G.K.)

**Keywords:** alarm photosynthesis, Antarctic, oxalate oxidase

## Abstract

Calcium oxalate (CaOx) crystals are widespread among plant species. Their functions are not yet completely understood; however, they can provide tolerance against multiple environmental stress factors. Recent evidence suggested that CaOx crystals function as carbon reservoirs since its decomposition provides CO_2_ that may be used as carbon source for photosynthesis. This might be advantageous in plants with reduced mesophyll conductance, such as the Antarctic plant *Colobanthus quitensis*, which have shown CO_2_ diffusion limitations. In this study, we evaluate the effect of two CO_2_ concentrations in the CaOx crystals decomposition and chlorophyll fluorescence of *C. quitensis*. Plants were exposed to airflows with 400 ppm and 11.5 ppm CO_2_ and the number and relative size of crystals, electron transport rate (ETR), and oxalate oxidase (OxO) activity were monitored along time (10 h). Here we showed that leaf crystal area decreases over time in plants with 11.5 ppm CO_2_, which was accompanied by increased OxO activity and only a slight decrease in the ETR. These results suggested a relation between CO_2_ limiting conditions and the CaOx crystals decomposition in *C. quitensis*. Hence, crystal decomposition could be a complementary endogenous mechanism for CO_2_ supply in plants facing the Antarctic stressful habitat.

## 1. Introduction

Calcium oxalate (CaOX) is a salt of oxalic acid (C_2_H_2_O_4_) and calcium (Ca^2+^) that forms insoluble crystals of diverse morphology [1]. Detected in at least 215 families, CaOx crystals are widespread among plant kingdom [2,3]. Crystals occur in roots, stems, leaves, flowers, fruits and seeds, and within epidermal, ground, and vascular tissues [4]. They are formed in the vacuoles of specialized cells called crystal idioblasts, which possess distinct structure and content from the surrounding cells. In leaves, crystals idioblasts are commonly located within mesophyll and/or bundle sheath extensions [5]. Some plants (including mainly succulents) like *Cactus senilis*, accumulate CaOx by as much as 85% by dry weight [6,7]. The huge variation in distribution among organs, tissues, and cells among plant species suggests that crystals may have independent origins of formation and multiple functions [8,9].

Recent evidence showed that indeed CaOx crystals represent multifunctional tools which are essential especially under stress conditions [8]. They are dynamic storage systems supplying calcium and oxalate ions upon demand. Both parts of these inclusions serve vital functions. The Ca part controls the levels of cytosolic concentration and immobilizes the excess quantities of this element, taking into account that plants do not have an excretory system. The oxalate part produced in the root can take part in nutrient acquisition, metal detoxification, mineral weathering, and selection of beneficial bacterial populations, whereas oxalate in the leaves can function as a dynamic carbon reservoir, providing CO_2_ in a process called alarm photosynthesis [10]. Moreover, oxalate of all organ and tissues can take part in defense reactions upon pathogen and/or herbivore attack [8,11]. 

Regarding alarm photosynthesis, CaOx crystals located within mesophyll, function as dynamic carbon reservoirs: Crystal decomposition releases CO_2_, which is further used for photosynthesis in plants exposed to CO_2_ limiting conditions, such as total or partial stomata closure during drought stress [10,12]. However, the function of CaOx as a source of CO_2_ for photosynthesis seems to be restricted to specific plant species or situations related to stressful environments, especially to water stress conditions [8,13]. The study of this process at the interspecific level in different climatic regions of the planet is still needed [10]. 

Antarctic is considered one of the territories with hardest conditions for plant species survival [14]. This is due to multiple extreme environmental traits of this ecosystem. In addition to the well-known low temperatures and sporadic high irradiance, plants inhabiting the Antarctic continent face short growing seasons, windiest climate, and high vapor pressure deficit, leading to leaf dryness [15]. Therefore, these plants become a proper model to research on this mechanism. 

*Deschampsia antarctica* Desv. (Poaceae) and *Colobanthus quitensis* (Kunth) Bartl. (Caryophyllaceae) are the only two plants that naturally have colonized parts of the maritime Antarctic [16]. *D. antarctica* is characterized by the absence of carbon calcium inclusions typical of the members of the Poaceae family [17,18], and therefore is not an appropriate candidate for this study. In contrast, *C. quitensis* has an abundant amount of CaOx crystals in its leaves. Moreover, *C. quitensis*, apart from its tolerance to extreme environmental conditions [19], represents a highly suitable plant model for the evaluation of the AP mechanism disposing three basic features that have been associated with the AP process: (1) a considerable amount of crystals with dimensions appropriate for accurate measurements of crystal properties (large idioblasts about 50 μm in diameter [17,18]), (2) the presence of transcripts with a high similarity to germin-like proteins (OxO enzymes) in the transcriptome [20,21], and (3) high CO_2_ diffusion limitations [22,23]. 

It has been shown that *C. quitensis* CO_2_ assimilation is highly limited by CO_2_ diffusion; this is partially due to leaf anatomical traits, such as mesophyll and chloroplast thickness [22]. This species has unusually low values of mesophyll conductance (g_m_) causing a constraint in the CO_2_ diffusion. Therefore, it becomes the principal restriction process for CO_2_ acquisition in the plant [23]. It is remarkable that this plant species can achieve high rates of photosynthesis with a very low g_m_. Authors suggest that some biochemical components might compensate this low CO_2_ diffusion, and therefore facilitate the CO_2_ availability, for instance, gas transport aquaporins or carbonic anhydrase and a robust enzymatic machinery [22,24]. Considering the recent findings regarding AP, an intriguing question arises: is this biochemical appendance of the photosynthetic machinery implicated in the photosynthetic function of *C. quitensis*?

For *C. quitensis*, AP might play a role as a complementary endogenous mechanism that could facilitate the supply of CO_2_, given the reported limitations in the diffusion of CO_2_. Therefore, this study aims to evaluate the dynamics of CaOx crystals in *C. quitensis* leaves under a CO_2_ limitation. We hypothesize that the exposure to a low external [CO_2_] causes decomposition of CaOx crystals in the leaves of *C. quitensis*, this will provide internal CO_2_ for a baseline level of photosynthesis, which will drain electrons from photosystem II even below the CO_2_ compensation point. The obtained results will give us novel data about CaOx crystals functions in plants and will add new evidence on the AP mechanism in a plant species from an extreme environment.

## 2. Results and Discussion

### 2.1. Calcium Oxalate Crystal Decomposition

**Figure 1:**
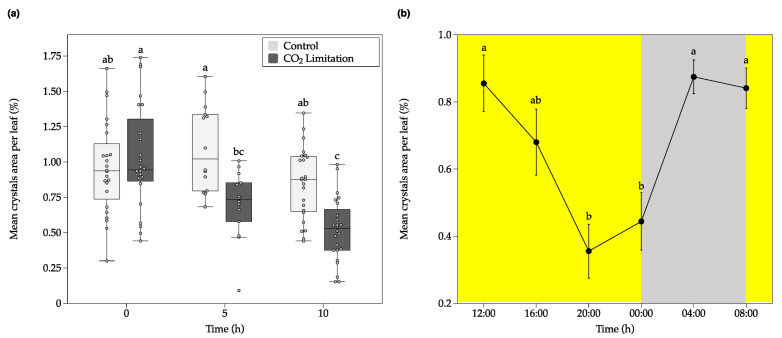
Fluctuations in total mean crystals area per leaf of *C. quitensis* plants under ambient or low CO_2_ concentration. (**a**), Boxplots representing the effect of the CO_2_ limitation treatment on leaf crystals area per leaf (%). The horizontal line indicates the mean and length of each whisker indicates the interquartile range (IQR); *n* = 14, different letters represent statistically significant differences (Two-way ANOVA; *p* < 0.05). In (**a**), plants were kept over time under constant light in airtight chambers injected either with ambient air (Control, 400 ppm CO_2_, white boxes) or filtered air with soda lime (CO_2_ limitation, 11.5 ppm CO_2_, gray boxes). (**b**), Diurnal fluctuations in CaOx crystal area in *C. quitensis* leaves. Plants were grown under optimal growth conditions. Yellow background denotes light hours, while gray background dark hours. Error bars denote SE of mean; *n* = 15. Different letters represent statistically significant differences between time of the day (one-way ANOVA; *p* < 0.05).

**Figure 2:**
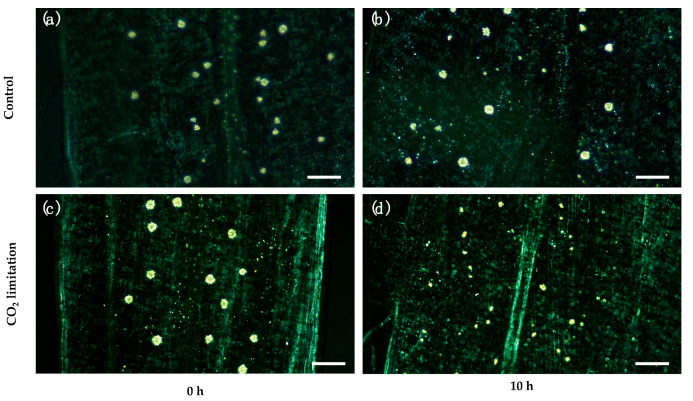
Paradermal view of the chlorine-bleached leaves under polarized light. CaOx crystals are visible as bright spots. Note the obvious differences in size and distribution of crystals between 0 h and 10 h under CO_2_ limitation. (**a**) Control leaf, time = 0 h, 400 ppm CO_2_. (**b**) Control leaf, time = 10 h, 400 ppm CO_2_. (**c**) Treatment leaf, time = 0 h, 11.5 ppm CO_2_. (**d**) Treatment leaf, time = 10 h, 11.5 ppm CO_2_. Bars = 200 μm.

A CO_2_ restriction experiment was performed with *C. quitensis* plants to test whether a CO_2_ limiting condition might trigger the decomposition of CaOx crystals. The experimental setup allowed to compare the CaOx crystal dynamics under a limiting CO_2_ condition on *C. quitensis* plants, with CO_2_ concentration close to 11.5 ppm, which is below the CO_2_ compensation point for this species (25–30 ppm) [22] and ambient 400 ppm CO_2_ (control). There were significant main effects for both CO_2_ concentration and time. There was a statistically significant interaction between CO_2_ concentration and time, where the relative area of crystals decreased as time passed under low [CO_2_]. Tukey’s post hoc test showed that, after ten hours of treatment, the mean of total crystal area per leaf was significantly lower in the 11.5 ppm-CO_2_-concentration group compared to the control group; a significant crystal decomposition was evident (Figure 1a, Figure 2 and Figure S1).

The imposed carbon limitation, generated by the low [CO_2_], triggered a significant reduction of CaOx crystals areas in the *C. quitensis* leaves, which is in accordance with our proposed hypothesis. The results are also in agreement with those reported by Tooulakou et al. (2016) [10], where a condition that boosted the stomatal closure and limited the availability of CO_2_ (e.g., exogenous application of abscisic acid or drought stress), increased the CaOX crystal decomposition in the leaves of *Amaranthus hybridus*. Furthermore, the number of crystals per leaf area decreased significantly in the low [CO_2_] treatment after 10 h (Figure S2). Therefore, the observed reduction of total crystal area per leaf (%) was the sum of both the reduction in size and number of crystals per leaf area. Consequently, given that complete crystal formation (maximum size) and maximum number of crystals per leaf are observed early in leaf development [25]; the observed differences in the crystals per leaf area should be a result of full crystal decomposition and not of differences in the number of idioblasts.

The CaOx crystal decomposition obtained here was also similar to that observed by Tooulakou et al. (2019) [26], where they showed that *A. hybridus* plants that were grown under CO_2_ restrictive conditions exhibited a considerable reduction in the leaf CaOx crystal volume over time, compared to the control group. Both Tooulakou et al. reports (2016; 2019) consider that leaf CaOx crystals act as dynamic carbon reservoirs, capable of providing CO_2_ for photosynthesis when the entry of atmospheric CO_2_ into the mesophyll is limited by an environmental factor. Therefore, the CaOx crystal decomposition that is observed during the day might be associated to carbon requirements for photosynthesis [10,12,26].

Moreover, the observation of the leaves crystals for 24 h, under optimal growth conditions, showed that these structures display diurnal fluctuations, similar to those reported in *A. hybridus* and *Oxalis corniculate* [12,27]. According to Figure 1b, during the first hours of light the mean crystal area per leaf kept stable. However, by 20:00 to 00:00 h the mean crystal area decreased significantly, while during the dark hours (00:00 to 08:00), these structures undergo a full recovery. During light time, and therefore the period where the plant is photosynthetically active, the crystals undergo a decomposition process. This process could be associated with supplementing—through the supply of subsidiary CO_2_ released from crystals—the CO_2_ requirement of this plant, since this species particularly suffers from a strong limitation in the acquisition of environmental CO_2_ [22]. 

Throughout dark hours, as there are no electrons (from light-dependent reactions) to fix carbon, the plants could restore the crystals. The recovery of the oxalate can occur through several metabolic pathways; however, its origin is exclusively biological [8]. Diurnal fluctuations evidence that crystals decomposition is not always associated to an environmental stress per se in *Colobanthus quitensis*, and more a complementary process that could be suppling subsidiary CO_2_ to its daily cycle. The obtained results suggest that the CaOX crystals in *C. quitensis* are a dynamic system, which respond to environmental stimuli, such as limitation of CO_2_ (Figure 1a), and fluctuates in a daily course (Figure 1b). 

### 2.2. Chlorophyll Fluorescence and Oxalate Oxidase Activity Measurements 

**Figure 3:**
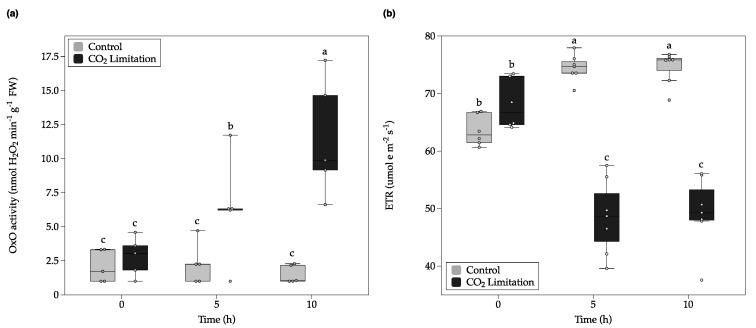
Fluctuations in the oxalate oxidase activity (OxO) (**a**), and electron transport rate (ETR) (**b**) of *C. quitensis* plants under ambient or low CO_2_ concentration. Plants were kept over time under constant light in airtight chambers injected either with ambient air (Control, 400 ppm CO_2_, white boxes) or filtered air with soda lime (CO_2_ limitation, 11.5 ppm CO_2_, gray boxes). The horizontal line indicates the mean and length of each whisker indicates the interquartile range (IQR); *n* = 5. Different letters represent statistically significant differences (two-way ANOVA; *p* < 0.05).

Tooulakou et al. (2016) also showed that crystal decomposition was accompanied by boosted oxalate oxidase enzymatic activity (OXO; transforms oxalate into CO_2_) [10]. The enzymatic analysis for oxalate oxidase activity on *C. quitensis* leaves showed that there were significant main effects for both CO_2_ concentration and Time, and also there was a statistically significant interaction between both effects on the oxalate oxidase activity. Tukey’s post hoc test showed that there were no significant differences between treatments at 0 h or 5 h of the test. However, statistically significant differences between the two studied groups were observed after 10 h (Figure 3a). The observed OXO activity was similar to that reported for *Podophyllum peltatum* after water stress [10]. The obtained results allow us to observe an association between the increase in oxalate oxidase activity and the decomposition of the CaOx crystals.

Chlorophyll fluorescence (ChlF) measurements showed that the electron transport rate (ETR) of the CO_2_-limited plants decreased significantly compared to the control group (Figure 3b). There were significant main effects for CO_2_ concentration, but not for Time. In addition, there was a statistically significant interaction between both effects on the ETR. However, despite the statistical differences, the plants exposed to low [CO_2_] for 10 h still maintain high ETR values (~50 μmol e m^−2^ s^−1^). This ETR values are similar to those reported before for this species under different temperatures and light intensity [22,28]. Following the AP hypothesis, a plant that does not have the AP mechanism and is exposed to a low [CO_2_], should experience an inhibition of photosynthesis also evident by a reduced ETR. It is known that in C3 plants exposed to low [CO_2_] the rate of carboxylation of Rubisco is reduced and consequently, the net photosynthetic rates are also affected due to substrate limitations; a situation that boosts photorespiration rates [29]. However, the obtained results showed just a 25% reduction of ETR in *C. quitensis* plants exposed to low [CO_2_] (Figure S3). 

The linearity between ETR and net CO_2_ assimilation is commonly absent in C3 species, especially due the existence of alternative electron sinks [30]. Furthermore, under excess light, reducing equivalents from photosynthetic electron transport (NADPH) are exported from the chloroplasts to the cytosol, via malate/oxaloacetate shuttle, and the mitochondrial non-phosphorylating pathways may facilitate the dissipation of these excess reductants in the cell [31]. Therefore, the occurrence of sufficient ETR values alone is not a satisfactory indication that AP is responsible for the use of electrons because there are other alternative electron sinks, such as photorespiration and mitochondrial respiratory chain. In *C. quitensis*, the photosynthetic electron transport is insensitive to variations in oxygen concentration under non-photorespiratory conditions, indicating that electron transport to oxygen (Mehler reaction) is negligible [32]. However, it has also been shown that under low CO_2_ availability, the relationship between ETR and Gross photosynthesis (A_G_) in *C. quitensis* leaves present high values, indicative of enhanced photorespiration rates [22].

### 2.3. Calcium Oxalate Crystal Decomposition under Non-Photorespiratory Conditions

**Figure 4:**
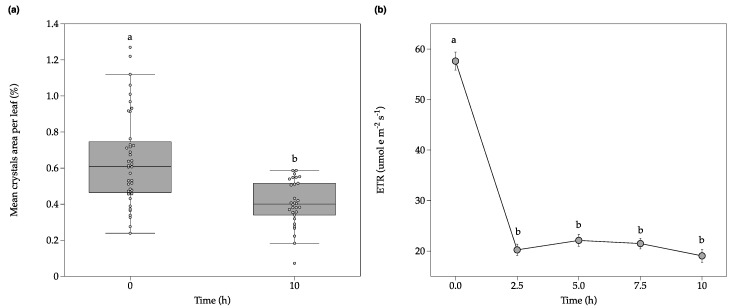
Fluctuations in total mean crystals area per leaf (**a**); and electron transport rate (ETR) (**b**) of *C. quitensis* plants under non-photorespiratory conditions (100% N_2_). Plants were kept over time under constant light in airtight chambers injected with N_2_ 100%. In (a), the horizontal line indicates the mean and length of each whisker indicates the interquartile range (IQR); *n* = 35; different letters represent statistically significant differences (Independent samples *t*-test, *p* < 0.01). In (**b**), error bars denote SE of mean; *n* = 15. Different letters represent statistically significant differences between time (One-way ANOVA; *p* < 0.05).

In order to eliminate the effect of photorespiration as an alternative electron sink on the observed level of ETR under the 11.5 ppm [CO_2_] treatment (50 μmol e m^−2^ s^−1^) (Figure 3b), as well to reduce the mitochondrial respiratory CO_2_ efflux, and the putative contribution of mitochondria electron chain consuming chloroplast redox power, a second individual experiment under non-photorespiratory conditions (100% N_2_) was performed. The *C. quitensis* plants showed a significant reduction in the crystals area per leaf between the beginning and the end of the treatment after 10 h under 100%-N_2_ atmosphere in the glass container (Figure 4a), as well as a high percentage of crystal decomposition (Figure S1). However, under non-photorespiratory conditions, the ETR values decreased intensely after 2 h of treatment but no further reduction was observed, and until the end of the treatment, the ETR values were kept constant close to ~20 μmol e m^−2^ s^−1^ (Figure 4b). The difference of ETR between low CO_2_ and 100% N_2_ was about 30 μmol e m^−2^ s^−1^, about 40% of total ETR; this is the putative contribution of photorespiration, mitochondrial respiration and Mehler reaction as alternative electron sinks to ETR in *C. quitensis* leaves. This is consistent with a high contribution of oxygen as an alternative electron sinks observed in other plant species [33,34]. Therefore, as previously reported by Saez et al. (2017) [22], it seems that photorespiration is enhanced in this species, which may help to counteract the harmful consequences that are generated as a result of a limited carbon assimilation. Photorespiration could also play a beneficial role in the dynamic and fast response of photosynthetic metabolism under CO_2_ limitations, as has been observed in *Arabidopsis thaliana* [35].

Despite having neither atmospheric CO_2_ nor O_2_ as final electron acceptors, *C. quitensis* plants were able to maintain a stable ETR value for about 10 h of stress. Theoretically, four electrons are required to reduce 2NADP^+^ to NADPH to fix one CO_2_ [36]. In practice, because not all the electron really flows linearly and alternative electron sinks are active, about 8–12 electrons are required per CO_2_ fixed. Therefore, with around ~20 μmol e m^−2^ s^−1^ of ETR, the *C. quitensis* plants could hypothetically fix about 2 μmol CO_2_ m^−2^ s^−1^, or even higher (about 5 μmol CO_2_ m^−2^ s^−1^) if we consider that under low oxygen the main alternative electron sinks, photorespiration, and Mehler reaction are constraint. If these CO_2_ molecules are effectively being supplied by the CaOx crystals decomposition needs to be probed. 

Alarm photosynthesis could be a process that may allow plants maintain a baseline level of photosynthesis when face stress situations. This mechanism is advantageous as a quenching regulator for the energy excess accumulated from the electron transport chain, when the light-dependent reactions are not in pace with photosynthetic CO_2_ assimilation from the atmosphere [10]. To this respect, it is possible that some unknown molecules could be involved as final electron acceptors in *C. quitensis* plants under a CO_2_ limiting condition, among them, the CO_2_ released from the CaOx crystals decomposition. 

The multiple functional role(s) of CaOx crystals in plants is not yet well understood and even some researchers have doubts about the functionality of the bioavailable calcium stocks in plants [13]. However, the obtained results generate a contribution on the understanding of the functions that have been attributed to CaOx crystals, and their relation with the AP mechanism, even though more evidence is still required to ensure that CO_2_ release by CaOx decomposition is being used for photosynthesis in *C. quitensis*. 

In this study, we found that *Colobanthus quitensis* plants exposed to a CO_2_ limitation significantly increased the CaOx crystal decomposition, as well as the oxalate oxidase activity in its leaves. This means that under stress conditions, the crystal decomposition could provide CO_2_ molecules to the mesophyll tissue [8]. In parallel, ETR decreased but remained stable when compared to the control group. Moreover, under non-photorespiratory conditions a significant CaOx crystal decomposition was also observed, whereas ETR decreased around 40% but was still adequate for the maintenance of a baseline level of photosynthesis if required. 

For the Antarctic plant *C. quitensis*, alarm photosynthesis could play an important role as a complementary endogenous mechanism that could facilitate a CO_2_ supply given the limitations in the CO_2_ diffusion that have been widely studied. Alarm photosynthesis is considered as a process that may enable the prevention of water losses when plants are under stressful conditions such as drought or strong winds coupled with low relative humidity. Further investigations on other extreme environment plant species is envisioned besides the genetic mechanisms of alarm photosynthesis, especially with focus on the CaOx genetic process of decomposition and regeneration (biosynthetic–degradation pathways), as well as the role of calcium ion during crystal recycling cycles, will allow a more detailed understanding of plant responses to intense drought scenarios. Future research should also focus on further understanding the diurnal fluctuations that have been observed in this plant, its relationship with daily hours and exposure to light. Likewise, attention should be paid to the description and characterization of the oxalate oxidase protein/gene and its regulation, not only because of their participation in the crystal decomposition processes, but also because of their association with stress tolerance processes.

## 3. Materials and Methods 

### 3.1. Plant Material and Growth Conditions

*Colobanthus quitensis* plants were collected in King George Island near to Henryk Arctowski Polish Antarctic station (62°09′34″ S; 58°28′19″ W) during March 2018. *C. quitensis* plants were reproduced vegetatively in plastic pots (5 × 5 × 5 cm) using a soil/peat/vermiculite mixture (3:1:2) and maintained in a greenhouse until having a regular-size cushion. Plants were fertilized with 0.2 g L^−1^ Phostrogen^®^ solution once a month. Before the experiment, *C. quitensis* plants were grown for at least 21 days (acclimation period) in a growth room (photoperiod 16/8 h, temperature 16 °C, photon flux density (photosynthetically active radiation) of 200 μmol m^−2^ s^−2^ and ambient air conditions (approximately 400 ppm CO_2_)). 

### 3.2. Experimental Design and Sample Collection

The individual plant cushions were placed inside a transparent airtight borosilicate container and were supplied with air (7.0 L/min) either at ambient (400 ppm, control) or low [CO_2_] (11.5 ppm, treatment) (Figure S4). A soda lime scrubber was used to achieve the low [CO_2_], which was continuously monitored by an infrared gas analyzer (IRGA-LI−6400XT, LI-COR Inc., Lincoln, NE, USA). During the experiment (10 h, from 08:00 to 18:00) plants were kept under 200 μmol m^−2^ s^−2^ PAR and 16 °C temperature. Leaf samples (24) were collected for crystal decomposition measurements. Additionally, 20 mg of leaves from 5 plants were collected at time 0, 5, and 10 h for oxalate oxidase activity determination. 

A second independent group of plants was kept at optimal growth conditions (temperature 16 °C, PAR of 200 μmol m^−2^ s^−2^ and ambient air conditions (approximately 400 ppm CO_2_)) for monitoring the leaves crystals for 24 h. Plants were exposed to 16 h of light (from 08:00 to 23:59) and 8 h of dark (00:00 to 07:59). Leaf samples (15) were collected for crystal decomposition measurements every four hours.

### 3.3. Chlorophyll Fluorescence

The electron transport rate (ETR) of *C. quitensis* plants exposed to both [CO_2_] was measured in vivo during the CO_2_-restrictive experiment. Chlorophyll fluorescence measurements were performed using a Maxi-Imaging-PAM Chlorophyll Fluorimeter (Walz, Effeltrich, Germany). Ten areas of interest (AOI) were selected from each cushion and the ETR was calculated according: ETR = Φ_PSII_·PAR·αL·(PSII/PSI); where Φ_PSII_ is the quantum efficiency of the photosystem II (PSII), PAR is the photosynthetically active radiation, αL the leaf absorptance (0.73 for control leaves, 0.68 for CO_2_ Limitation), and PSII/PSI the distribution of absorbed energy between the two photosystems (assumed to be 0.5). Φ_PSII_ was measured with a Saturation Pulse (6000 μmol photons m^−2^ s^−1^, 800 ms) applied after 3 min illumination at 750 μmol quanta m^−2^ s^−1^ of actinic light (AL) (Figure S5). The leaf absorptance was directly measured in the plants using the Maxi-Imaging PAM as described by Saéz et al., 2018 [37]. Briefly, successive illumination of the samples with red (R) and near infrared (NIR) light and the capture of each remission image were used by the equipment software to calculate pixel by pixel as follows: Abs = 1 − R/NIR. The minimum number of cushions used for the analysis (*n*) was at least 5.

### 3.4. Measurements of Crystal Degradation

The collected *C. quitensis* leaves were bleached in sodium hypochlorite solution (5% p/p) according to Tooulakou et al. (2016) [10]. Briefly, whole mature leaves were put in an aqueous solution of commercial bleach for 48 h until full depigmentation. Depigmented leaves were rinsed with abundant distillated water and then put between two microscope slides. Samples were observed under an optical microscope adapted with a polarizing filter at 10× magnification Leica DM750-Camera Leica ICC50W (Leica Microsystems, Wetzlar, Hesse, Germany). Several images were taken per leaf, covering the total leaf area. The area of each crystal was calculated by digital image analysis (ImageJ-Fiji v 2.0.0-rc69/1.52i) [38]. For each individual leaf, several images were taken comprising the total leaf area. Each individual image was analyzed as follows: (1) image was converted to 8 bits, (2) 8 bits image was converted to Mask, and (3) the tool “Analyzing Particles” was used to count and measure all crystals area in the picture using the following parameters: Size 400–5000 pixel^2^, Circularity 0.35–1.00. The total counts (crystals area) of all images from an individual leaf were sum together to obtain the total area of crystals per leaf. Then, this value is divided by the total leaf area to obtain a ratio area crystals/area leaf. The minimum number of leaves used for the analysis at each time (*n*) was at least 14. 

### 3.5. Oxalate Oxidase Activity Determination

The activity of the oxalate oxidase enzyme was determined at time 0, 5, and 10 h of the CO_2_ restrictive experiment using the Oxalate Oxidase Activity Assay Kit (BioVision, Inc., Milpitas, CA, USA). Assays were performed following kit protocol using 20 mg of *C. quitensis* fresh leaves from 5 plants (*n* = 5). In this test, the decomposition of oxalate by an oxalate oxidase release hydrogen peroxide, which generates a fluorescent signal directly proportional to the amount of active oxalate oxidase present in samples.

### 3.6. Statistical Analysis

Two-way analysis of variance (ANOVA) at a 95% level of significance (*p* < 0.05) were applied using JASP software (Version 0.13.1) [39] to assess the effects of both time and CO_2_ concentration. Tukey Post Hoc Test was carried out in those cases where ANOVA was significant. One-way ANOVA was used to analyze one factor multiple comparation and Independent samples *t*-test were applied for simple comparison. The assumption of data normality, and homoscedasticity were tested with the Shapiro–Wilk and Levenes’s test (data regarding all the statistical tests can be found in Tables S1–S8)

## Supplementary Materials

The following are available online at https://www.mdpi.com/2223-7747/9/10/1307/s1, Figure S1: Decomposition percentage of CaOx crystals in *C. quitensis* leaves under adequate (Control, 400 ppm CO_2_), low CO_2_ (CO_2_ limitation, 11 ppm CO_2_) and non-photorespiratory conditions (100% N_2_, 2 ppm CO_2_) at the end of the treatment. The horizontal line indicates the mean and length of each whisker indicates the interquartile range (IQR); *n* = 5. Different letters denote significant differences between groups (one-way ANOVA; *p* < 0.05). Figure S2: CaOx crystals number counted in whole *C. quitensis* leaves under adequate (Control, 400 ppm CO_2_) or low (Treatment, 11 ppm CO_2_) CO_2_ concentration. The horizontal line indicates the mean and length of each whisker indicates the interquartile range (IQR); *n* = 14. Different letters represent statistically significant differences (two-way ANOVA; *p* < 0.05). Figure S3: Light responses curves (ETR/ETR_max_ %) of *Colobanthus quitensis* under adequate (Control, 400 ppm CO_2_) and low CO_2_ (CO_2_ limitation, 11 ppm CO_2_). Measurements were performed at 16 °C. Error bars denote SD of mean; *n* = 6. Figure S4: Experimental setup: System used to run the CO_2_ restriction experiment on *C. quitensis* plants. Figure S5: Quantum yield of PSII (φPS II) in *C. quitensis* leaves under adequate (Control, 400 ppm CO_2_) or low (Treatment, 11 ppm CO_2_) CO_2_ concentration. Error bars denote SD of mean; *n* = 15. Table S1: Two-way independent ANOVA results for Figure 1a data. Table S2: One-way independent ANOVA results for Figure 1b data. Table S3: Two-way independent ANOVA results for Figure 3a data. Table S4: Two-way independent ANOVA results for Figure 3b data. Table S5: Independent samples *t*-test results for Figure 4a data. Table S6: One-way independent ANOVA results for Figure 4b data. Table S7: One-way independent ANOVA results for Figure S1 data. Table S8: Two-way independent ANOVA results for Figure S2 data.
