# Supplementary material for: Decomposition of Calcium Oxalate Crystals in Colobanthus quitensis under CO2 Limiting Conditions
Source: Plants (Basel). 2020 Oct 2;9(10):1307. doi: 10.3390/plants9101307 (PMC7600318; doi:10.3390/plants9101307)
Supplement: Supplementary file 1 [file plants-09-01307-s001.pdf]

plants

MDPI

Supplementary materials

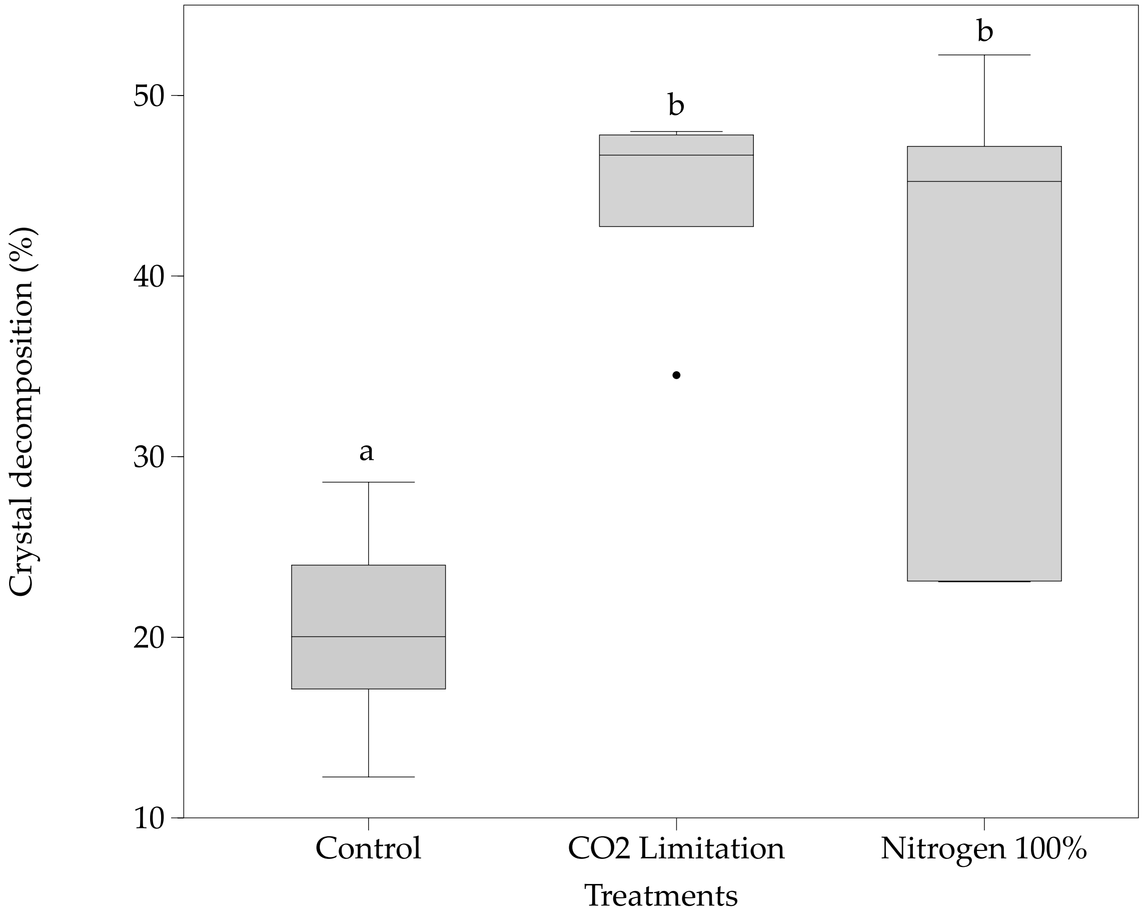

Figure S1. Decomposition percentage of CaOx crystals in *C. quitensis* leaves under adequate (Control, 400 ppm CO_2), low CO_2 (CO_2 limitation, 11 ppm CO_2) and non-photorespiratory conditions (100 % N_2, 2 ppm CO_2) at the end of the treatment. The horizontal line indicates the mean and length of each whisker indicates the interquartile range (IQR); n = 5. Different letters denote significant differences between groups (One-way ANOVA; $p < 0.05$).

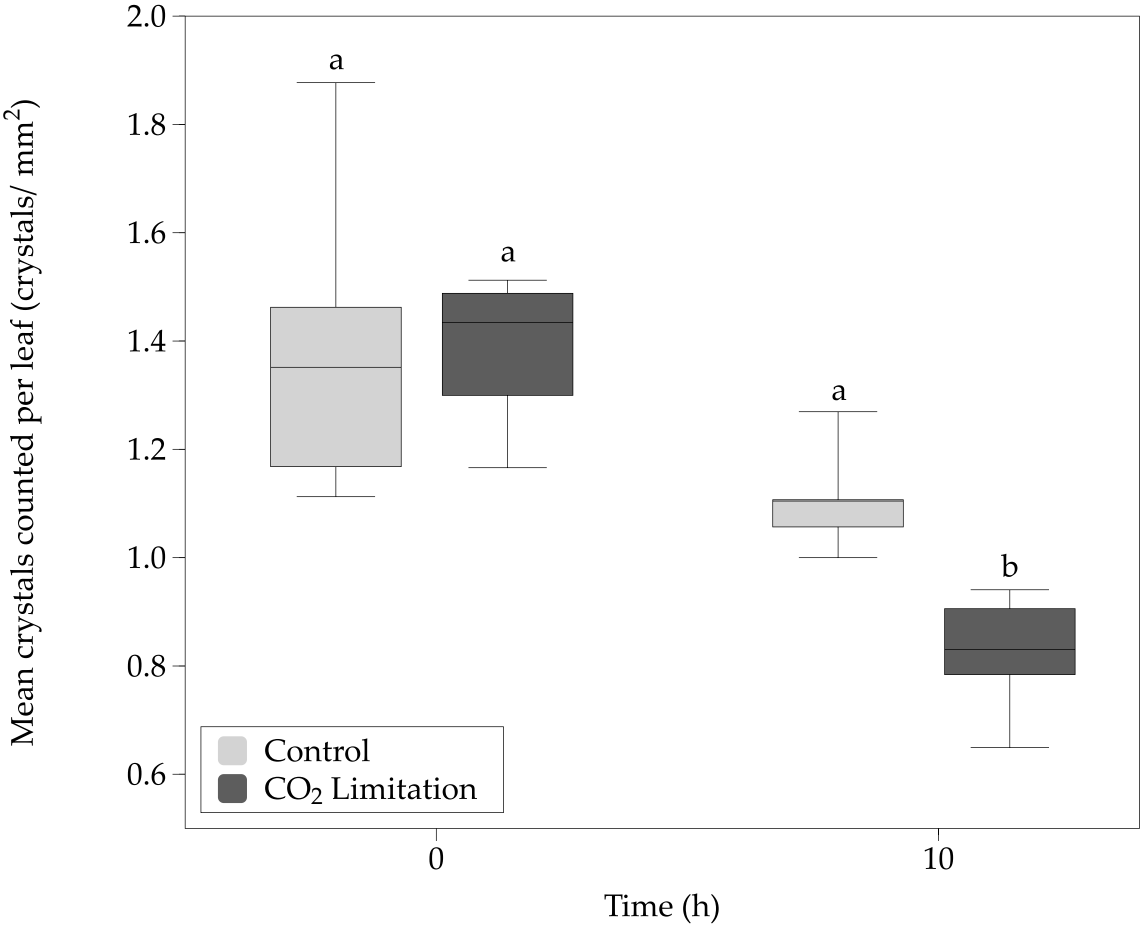

Figure S2. CaOx crystals number counted in whole *C. quitensis* leaves under adequate (Control, 400 ppm CO_2) or low (Treatment, 11 ppm CO_2) CO_2 concentration. The horizontal line indicates the mean and length of each whisker indicates the interquartile range (IQR); n = 14, different letters represent statistically significant differences (Two-way ANOVA; $p < 0.05$).

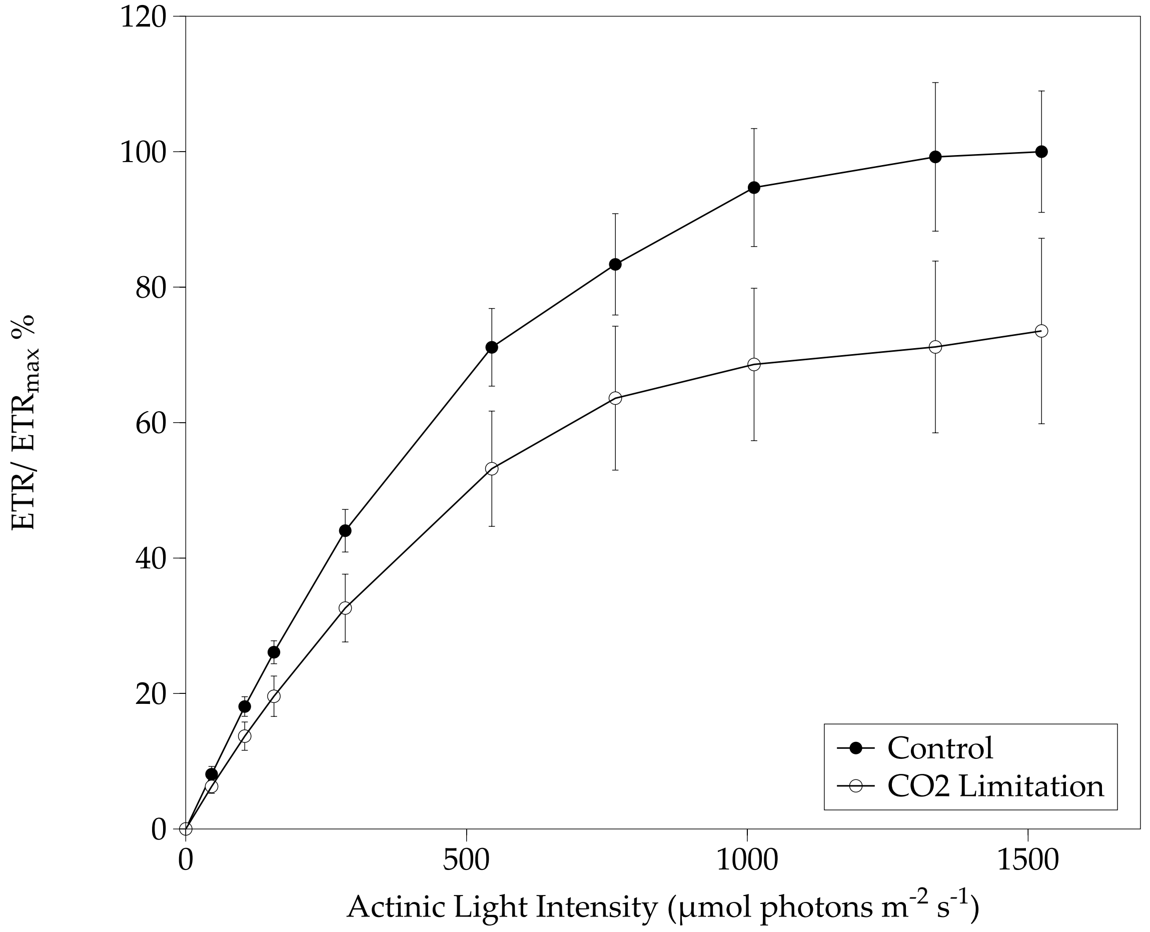

Figure S3. Light responses curves (ETR/ETR_{max} %) of *Colobanthus quitensis* under adequate (Control, 400 ppm CO_2) and low CO_2 (CO_2 limitation, 11 ppm CO_2) concentrations. Measurements were performed at 16 °C. Error bars denote SD of mean; n = 6.

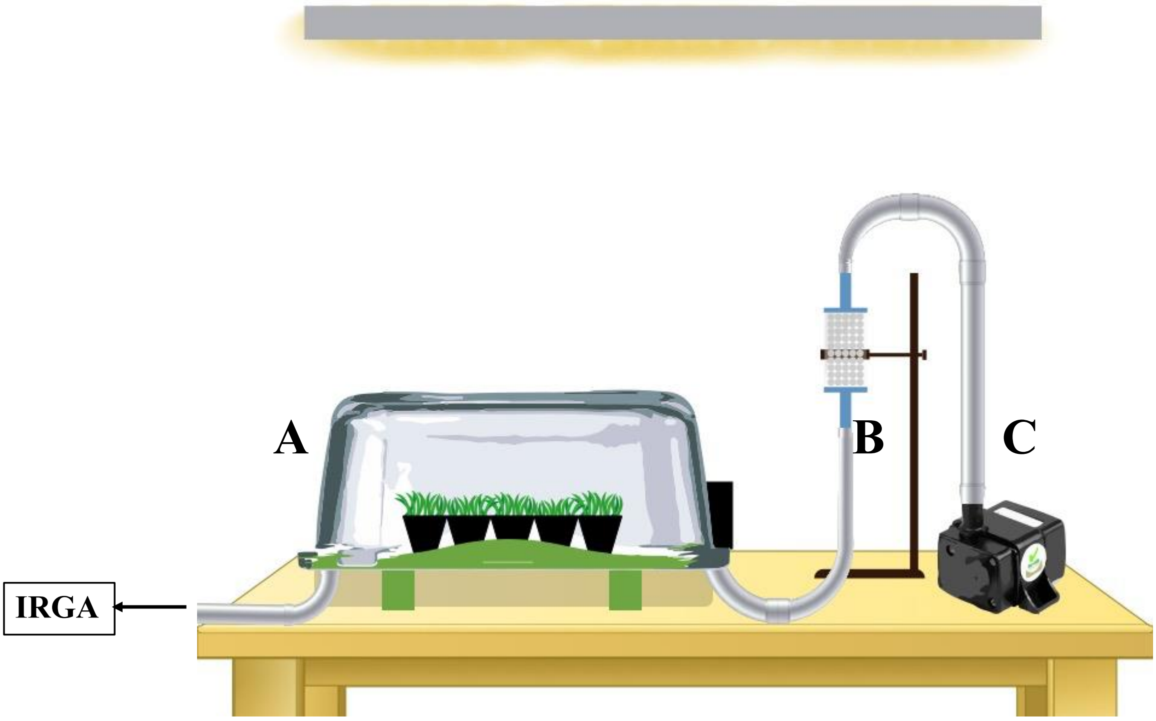

Figure S4. Experimental setup. System used to run the CO_2 restriction experiment on *C. quitensis* plants. A, Airtight container. B, Soda lime scrubber. C, Air pump. IRGA, Infra-Red Gas Analyzer ((RGA-LI-6400XT, LI-COR Inc., Lincoln, NE, USA).

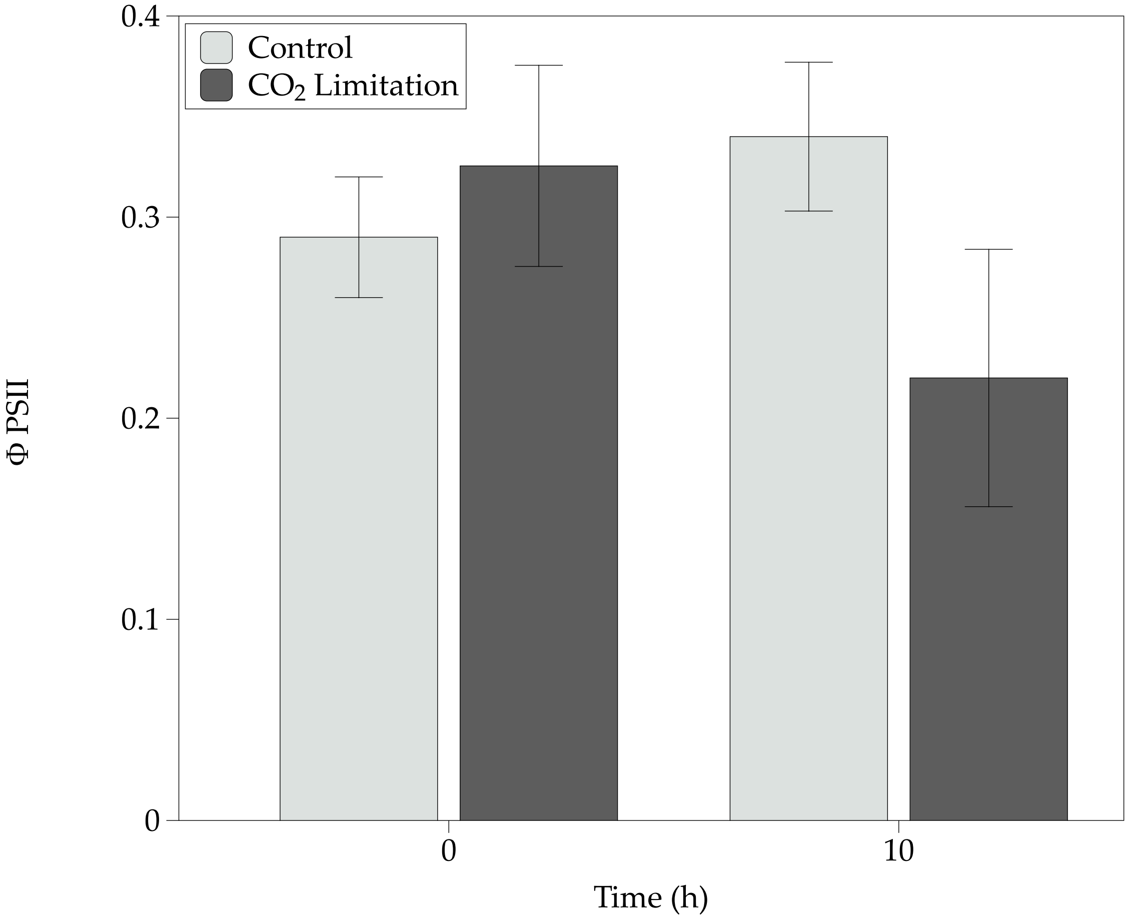

Figure S5. Quantum yield of PSII (φPS II) in *C. quitensis* leaves under adequate (Control, 400 ppm CO_2) or low (Treatment, 11 ppm CO_2) CO_2 concentration. Error bars denote SD of mean; n = 15.

Table S1. Two-way independent ANOVA results for Figure 1 (a) data.

ANOVA – Mean crystal area per leaf (%).

Cases	Sum of Squares	df	Mean Square	F	p	ω^2
Group	1.398	1	1.398	16.069	< .001	0.084
Time	2.412	2	1.206	13.861	< .001	0.144
Group ✻ Time	1.370	2	0.685	7.874	< .001	0.077
Residuals	10.266	118	0.087			

Note. *Type III Sum of Squares*

Post Hoc Comparisons - Group ✻ Time Tukey Standard

			99% CI for Mean Difference				
		Mean Difference	Lower	Upper	SE	t	p_{tukey}
CO2 limitation 0 h	Control 0 h	0.072	-0.221	0.366	0.085	0.849	0.958
	CO2 limitation 5 h	0.333	-0.009	0.675	0.099	3.357	0.013 *
	Control 5 h	-0.066	-0.408	0.276	0.099	-0.662	0.986
	CO2 limitation 10h	0.514	0.220	0.807	0.085	6.036	< .001 ***
	Control 10	0.183	-0.111	0.476	0.085	2.144	0.272
Control 0 h	CO2 limitation 5h	0.261	-0.081	0.603	0.099	2.629	0.098
	Control 5 h	-0.138	-0.480	0.204	0.099	-1.390	0.733
	CO2 limitation 10h	0.442	0.148	0.735	0.085	5.187	< .001 ***
	Control 10 h	0.110	-0.183	0.404	0.085	1.295	0.787
CO2 limitation 5 h	Control 5 h	-0.399	-0.783	-0.014	0.111	-3.576	0.007 **
	CO2 limitation 10h	0.181	-0.161	0.523	0.099	1.824	0.455
	Control 10	-0.150	-0.492	0.191	0.099	-1.517	0.654
Control, 5	CO2 limitation 10h	0.580	0.238	0.921	0.099	5.843	< .001 ***
	Control 10 h	0.248	-0.094	0.590	0.099	2.502	0.132
CO2 limitation 10 h	Control 10 h	-0.331	-0.625	-0.038	0.085	-3.892	0.002 **

Note. * p < .05, ** p < .01, *** p < .001

Table S2. One-way independent ANOVA results for Figure 1 (b) data.

ANOVA - Mean crystal area per leaf (%).

Cases	Sum of Squares	df	Mean Square	F	p
Grupo	7.683e -5	5	1.537e -5	9.448	< .001
Residual	1.952e -5	12	1.626e -6		

Note. Type III Sum of Squares

Post Hoc Comparisons – Group Tukey Standard

		Mean Difference	SE	t	p_{tukey}
00:00:00	04:00:00	-0.004	0.001	-4.138	0.013
	08:00:00	-0.004	0.001	-3.813	0.023
	12:00:00	-0.004	0.001	-3.950	0.019
	16:00:00	-0.002	0.001	-1.955	0.418
	20:00:00	8.852e -4	0.001	0.850	0.951
04:00:00	08:00:00	3.383e -4	0.001	0.325	0.999
	12:00:00	1.955e -4	0.001	0.188	1.000
	16:00:00	0.002	0.001	2.183	0.312
	20:00:00	0.005	0.001	4.988	0.003
08:00:00	12:00:00	-1.428e -4	0.001	-0.137	1.000
	16:00:00	0.002	0.001	1.858	0.468
	20:00:00	0.005	0.001	4.663	0.006
12:00:00	16:00:00	0.002	0.001	1.995	0.398
	20:00:00	0.005	0.001	4.801	0.005
16:00:00	20:00:00	0.003	0.001	2.805	0.124

Table S3. Two-way independent ANOVA results for Figure 3 (a) data.

ANOVA - Oxo_Activity

Cases	Sum of Squares	df	Mean Square	F	p
Time	82.595	2	41.297	6.318	0.006
Group	182.741	1	182.741	27.956	< .001
Time ✻ Group	110.197	2	55.098	8.429	0.002
Residuals	156.880	24	6.537		

Note. Type III Sum of Squares

Post Hoc Comparisons - Time ✻ Group Tukey Standard

		Mean Difference	SE	t	p_{tukey}	
CO2 limitation 0h	CO2 limitation 5h	-3.500	1.617	-2.165	0.290	
	CO2 limitation 10h	-8.692	1.617	-5.375	< .001	***
	Control 0h	0.741	1.617	0.458	0.997	
	Control 5h	0.561	1.617	0.347	0.999	
	Control 10h	1.315	1.617	0.813	0.962	
CO2 limitation 5h	CO2 limitation 10h	-5.191	1.617	-3.211	0.039	*
	Control 0h	4.241	1.617	2.623	0.130	
	Control 5h	4.061	1.617	2.512	0.160	
	Control 10h	4.815	1.617	2.978	0.064	
CO2 limitation 10h	Control 0h	9.432	1.617	5.833	< .001	***
	Control 5h	9.253	1.617	5.722	< .001	***
	Control 10h	10.007	1.617	6.188	< .001	***
Control 0h	5 Control 5h	-0.179	1.617	-0.111	1.000	
	Control 10h	0.575	1.617	0.355	0.999	
Control 5h	Control 10h	0.754	1.617	0.466	0.997	

* p < .05, ** p < .01, *** p < .001

Table S4. Two-way independent ANOVA results for Figure 3 (b) data.

ANOVA - ETR

Cases	Sum of Squares	df	Mean Square	F	p	ω^2
Time	139.466	2	69.733	3.208	0.054	0.019
Group	2277.158	1	2277.158	104.761	< .001	0.452
Time ✻ Group	1857.980	2	928.990	42.739	< .001	0.363
Residuals	695.571	32	21.737			

Note. Type III Sum of Squares

Post Hoc Comparisons - Time ✻ Group Tukey Standard

		Mean Difference	SE	t	p_{tukey}	
CO2 limitation 0h	CO2 limitation 5h	19.580	2.594	7.549	< .001	***
	CO2 limitation 10h	18.755	2.594	7.230	< .001	***
	Control 0h	4.542	2.692	1.687	0.550	
	Control 5h	-6.537	2.692	-2.429	0.177	
	Control 10h	-6.239	2.692	-2.318	0.217	
CO2 limitation 5h	CO2 limitation 10h	-0.826	2.492	-0.331	0.999	
	Control 0h	-15.038	2.594	-5.798	< .001	***
	Control 5h	-26.117	2.594	-10.069	< .001	***
	Control 10h	-25.820	2.594	-9.954	< .001	***
CO2 limitation 10h	Control 0h	-14.213	2.594	-5.479	< .001	***
	Control 5h	-25.292	2.594	-9.751	< .001	***
	Control 10h	-24.994	2.594	-9.636	< .001	***
Control 0h	5 Control 5h	-11.079	2.692	-4.116	0.003	**
	Control 10h	-10.781	2.692	-4.005	0.004	**
Control 5h	Control 10h	0.298	2.692	0.111	1.000	

* p < .05, ** p < .01, *** p < .001

Table S5. Independent Samples T-Test results for Figure 4 (a) data.

Independent Samples T-Test

	t	df	p
Mean crystal area per leaf (%)	-3.544	75	< .001

Note. Student's t-test.

Table S6. One-way independent ANOVA results for Figure 4 (b) data.

ANOVA - ETR

Cases	Sum of Squares	df	Mean Square	F	p
Time	54953.654	4	13738.413	156.153	< .001
Residuals	17332.193	197	87.981		

Note. *Type III Sum of Squares*

Post Hoc Comparisons – Time Tukey Standard

		Mean Difference	SE	t	p_{tukey}
0	2.5	37.396	1.997	18.727	< .001 ***
	5	35.520	1.859	19.106	< .001 ***
	7.5	36.149	1.895	19.080	< .001 ***
	10	38.575	2.438	15.824	< .001 ***
2.5	5	-1.876	2.087	-0.899	0.897
	7.5	-1.247	2.119	-0.588	0.977
	10	1.179	2.616	0.451	0.991
5	7.5	0.629	1.990	0.316	0.998
	10	3.054	2.512	1.216	0.742
7.5	10	2.425	2.539	0.955	0.875

Note. *** p < .001

Table S7. One-way independent ANOVA results for Figure S1 data.

ANOVA – Decomposition %

Cases	Sum of Squares	df	Mean Square	F	p
Goup	1513.397	2	756.699	8.449	0.005
Residuals	1074.671	12	89.556		

Note. Type III Sum of Squares

Post Hoc Comparisons - Group

		Mean Difference	SE	t	p_{tukey}	
CO2, Limitation	Control	23.602	5.985	3.943	0.005	**
	Nitrogen, 100%	5.782	5.985	0.966	0.611	
Control	Nitrogen, 100%	-17.820	5.985	-2.977	0.029	*

Note. * $p < .05$, ** $p < .01$

Table S8. Two-way independent ANOVA results for Figure S2 data.

ANOVA - CaOx crystals number counted

Cases	Sum of Squares	df	Mean Square	F	p	ω^2
Group	0.161	1	0.161	4.364	0.052	0.070
Time	0.822	1	0.822	22.333	< .001	0.441
Group ✻ Time	0.135	1	0.135	3.681	0.072	0.055
Residuals	0.626	17	0.037			

Note. Type III Sum of Squares

Post Hoc Comparisons - Group ✻ Time – Tukey Standard

		Mean Difference	SE	t	p_{tukey}	
CO2, limitation, 0	Control, 0	-0.014	0.121	-0.118	0.999	
	CO2, limitation, 10	0.558	0.121	4.599	0.001	**
	Control, 10	0.221	0.116	1.906	0.262	
Control, 0	CO2, limitation, 10	0.572	0.121	4.717	0.001	**
	Control, 10	0.236	0.116	2.030	0.216	
CO2, limitation, 10	Control, 10	-0.337	0.116	-2.897	0.045	*

* $p < .05$, ** $p < .01$
